# Could it be osteoarthritis? How dog owners and veterinary surgeons describe identifying canine osteoarthritis in a general practice setting

**DOI:** 10.1016/j.prevetmed.2020.105198

**Published:** 2020-12

**Authors:** Zoe Belshaw, Rachel Dean, Lucy Asher

**Affiliations:** School of Veterinary Medicine and Science, University of Nottingham Sutton Bonington Campus, Loughborough, Leicestershire, LE12 5RD, UK

**Keywords:** Canine osteoarthritis, Veterinary, Owner, Qualitative research, Thematic analysis, Diagnostic testing

## Abstract

Canine osteoarthritis is a common, painful condition that is typically managed in a general practice setting. Osteoarthritis may have significant negative impacts on the welfare of both dogs and their owners. Anticipated early clinical signs of canine osteoarthritis and the preferred route to its diagnosis are well described by veterinary subject experts in published literature. However, little is known about changes owners first recognise in a dog ultimately diagnosed with osteoarthritis, how they make decisions about when to present that dog to a general practitioner, or whether the described diagnostic pathways are followed by general practitioners. The aim of this research was to investigate how dog owners and veterinary surgeons describe identifying and diagnosing canine osteoarthritis.

Owners of osteoarthritic dogs were recruited for semi-structured interview, and veterinary surgeons working in general practice were invited to take part in practice-based focus groups. Transcripts from both datasets were thematically analysed using a contextualist epistemology with an ontology based on critical realism to construct convergent themes from latent and semantic codes. Thirty-two interviews were completed with 40 owners from 32 households who discussed 35 dogs with osteoarthritis, and 26 veterinary surgeons engaged in four practice-based focus groups.

Owners described identifying a wide range of acute and chronic, typically subtle and intermittent, behavioural and demeanour changes prior to their dogs’ osteoarthritis diagnosis. Few attributed these changes to canine osteoarthritis, and some waited many months before presenting their dog to a veterinary practice. Veterinary surgeons described a consistent ‘typical osteoarthritis’ presentation that they recognised through history taking and clinical examination. Their diagnostic work-up rarely followed that advocated by subject experts for reasons including lack of time and perceptions that it would not change the outcome. Many veterinary surgeons described frustration that some owners did not accept their recommendations to provide analgesia for affected dogs. Short consultation lengths, poor awareness of owner knowledge levels, and lack of recognition of the importance of owners’ prior knowledge, beliefs and assumptions may contribute to these consultations being perceived as challenging by some veterinary surgeons and owners.

This research demonstrates that veterinary surgeons and owners want dogs with clinical signs of osteoarthritis to be happy and comfortable, but that ineffective communication and lack of trust in the consulting room may be a barrier. Our data identifies many new avenues for future research and improved communication strategies that could facilitate earlier identification and treatment of canine osteoarthritis in general practice.

## Highlights

• Owners describe a range of early behavioural indicators of canine osteoarthritis.
• Their beliefs and prior knowledge may impact when and how they seek advice.
• Vets in general practice describe a common “typical osteoarthritis” presentation.
• History, examination and trial treatment are used to diagnose osteoarthritis.
• Vets and owners may find osteoarthritis consultations frustrating and unrewarding.

## Graphical abstract

**Figure:**
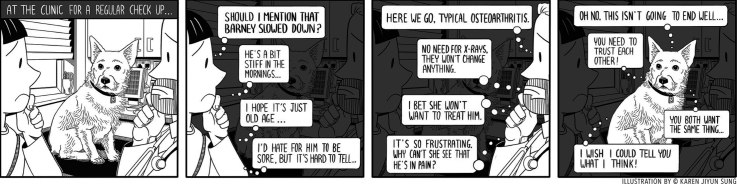

## 1 Introduction

Canine osteoarthritis is a common disease which may significantly compromise the welfare of affected dogs (Anderson et al., 2018; Belshaw and Yeates, 2018; Summers et al., 2019). Recent research from this group has also identified that canine osteoarthritis can have a substantial impact on the welfare of owners, through impacts including reduced dog walking distance, speed and location variety, and stress related to their dog’s diagnosis and how to optimise disease management (Belshaw et al., 2020a, 2020b). There are effective interventions to mitigate affected dogs’ pain and mobility problems (Sanderson et al., 2009), but dog ownership practices render dogs reliant on their human carers to provide for their needs (Glanville et al., 2020). Dog owners should be motivated, and may feel morally obliged, to promptly act upon any physical or behavioural changes identified (Glanville et al., 2020). However, a range of barriers may exist. Owners could be unaware that osteoarthritis affects dogs or might struggle to recognise relevant behavioural indicators. Pain behaviours may be confused with a behaviour problem (Mills et al., 2020), and some dog breeds are perceived by the public to be less pain sensitive than others (Gruen et al., 2020). Owners who are aware of a problem may delay their dog’s presentation to a veterinary practice if they are uncertain or fearful of the veterinary surgeon’s recommended actions (Belshaw et al., 2016a; Christiansen et al., 2016). In human healthcare, disincentives for people with joint pain to seek medical advice include beliefs that stiffness and osteoarthritis are normal ageing changes (Paskins et al., 2013, 2014), and that the condition is untreatable (Cuperus et al., 2013). This is likely to be amplified by the lack of societal awareness of this disease which can mask its incidence and impact (Versus Arthritis, 2019). Contextual factors, values, attitudes and beliefs have been described as influencing the decisions of owners of horses with colic (Scantlebury et al., 2014), and in relation to the emerging concept of dog owner duty of care (Glanville et al., 2020), but have not been explored in relation to canine osteoarthritis.

To access prescription medication for canine osteoarthritis, the affected dog needs to be presented to a veterinary surgeon to confirm the diagnosis. The pathway for canine osteoarthritis diagnosis and management by veterinary surgeons is well described in the published literature by subject experts. An accurate, complete clinical history may elicit evidence of inactivity stiffness, reluctance to exercise, difficulty jumping and exercise intolerance (Cachon et al., 2018; Innes, 2012; Pettit and German, 2015). Tools or clinical metrology instruments such as the Canine Brief Pain Inventory can further characterise these changes (e.g. Brown et al., 2008; Brown, 2014; Cachon et al., 2018; Walton et al., 2013). A complete physical examination is suggested, with mobility and gait assessments (Pettit and German, 2015). Subsequent radiography has been described the “mainstay” of diagnostic tests to ensure that any underlying primary joint abnormality has been identified (Cachon et al., 2018; Innes, 2012; Pettit and German, 2015). A plethora of tools including computerised tomography, forceplate gait analysis and arthroscopy may then be used to confirm the presence of the disease and assess severity (Belshaw et al., 2016b; Pettit and German, 2015; Walton et al., 2018). The clinical predictive value of the individual components of this pathway for a diagnosis of canine osteoarthritis has not been established; neither have the clinical decision thresholds at which treatment would be instigated (Guervara et al., 2019).

Research has not previously explored whether this expert opinion is followed by, or suitable for, general practitioners who are responsible for the majority of diagnoses of canine osteoarthritis (Belshaw et al., 2016b; van Cleven et al., 2018). Variance in decisions and treatments between general practitioners and specialists have been described in veterinary ophthalmology (White et al., 2018a, 2018b) and cardiology (Davies et al., 2015). The reasons for this variance may be multifactorial since general practice consultations often cover a wide range of problems and co-morbidities (Robinson et al., 2015a) whereas specialists are likely to focus on a single problem. In human healthcare, challenges of keeping up to date as a general practitioner, uncertainty about which aspects of conflicting expert advice should be followed, and pressure from both colleagues and patients to act in certain ways are acknowledged barriers to following expert guidelines (Ingvarsson et al., 2020). Veterinary general practice consultations are also time limited which may compromise history taking and in-consultation diagnostic testing (Belshaw et al., 2018a; Robinson et al., 2015b). These factors may be pertinent to the diagnosis of canine osteoarthritis in general practice, where lameness is often a non-presenting problem, defined as something that is not the primary reason for the dog’s appointment with the veterinary surgeon (Belshaw et al., 2018a; Robinson et al., 2014).

A growing body of quantitative (German et al., 2010; O’Neill et al., 2013; Robinson et al., 2016; Singleton et al., 2019) and qualitative research (Belshaw et al., 2016a; Belshaw et al., 2018a, 2018b, 2018c, 2018d; Belshaw et al., 2020a, b; King et al., 2018) describes how general practitioners and pet owners make decisions. Qualitative research has not previously been published that explores the recognition and diagnosis of canine osteoarthritis. Since this diagnostic process will involve observations and decisions made by dog owners and veterinary surgeons, separately and together, both parties need to be involved in the research. The aim of this study was therefore to investigate how dog owners and veterinary surgeons describe identifying and diagnosing canine osteoarthritis. The objective was to conduct interviews with dog owners and focus groups with veterinary surgeons working in general practice in the United Kingdom to explore how canine osteoarthritis is recognised and diagnosed.

## 2 Methods

Data presented are from a qualitative study using interviews with dog owners and focus groups with veterinary surgeons and veterinary nurses to explore their experiences of managing dogs with osteoarthritis. Veterinary nurse data are not included since veterinary nurses are unable to diagnose canine osteoarthritis or instigate prescription treatments. This is one of a series of publications from a significant piece of research (Belshaw et al., 2016a, 2016b, 2020a, 2020b; Ladha et al., 2017). Reporting follows the Consolidated Criteria for Reporting Qualitative Research (COREQ; Tong et al., 2007). The reporting quality of the thematic analysis described in this publication can also be compared against the recently published checklist tool included in Braun and Clarke (2020a). Ethical approval for this research was obtained from the School of Veterinary Medicine and Science, University of Nottingham (Reference: 1106 140310).

### 2.1 Dog owner interview data collection

Interviews were chosen as a data collection method for owners to enable the exploration of detailed, chronological narratives that would not have been possible through focus groups or questionnaires. Interview recruitment was based on a purposive sampling frame of owner, dog and location factors constructed by the authors (see Belshaw et al., 2020a for full details). This intended to capture the widest possible range of owner experiences. Inclusion criteria for interviewees were: a) ownership of a dog at least 5 years of age treated or managed for osteoarthritis in at least one limb; AND b) residency of dog and owner(s) in the UK; AND c) availability for interview during the study period. It was not necessary that the dog’s osteoarthritis had been confirmed by any specific diagnostic test, just that the owner had been advised by their veterinary surgeon that their dog was affected.

Interviewees were recruited by displaying posters in a convenience sample of 10 veterinary practices in England and Scotland, by snowball sampling or from the authors’ networks. Incentives to participate were not provided. Interested owners were sent information about the background and purpose of the study including details of the interviewer (ZB)’s identity as a veterinary surgeon and owner of an osteoarthritic dog. An interview date was arranged if they were eligible and willing to participate. Written consent to participate was confirmed prior to interview, and interviewees were advised that they could withdraw from the study at any time.

Interviews were conducted by ZB in owners’ homes between February and August 2014. A semi-structured interview guide was used to ensure the same broad topics were covered (see Belshaw et al., 2020a for a copy), but owners were encouraged to lead the interview. Topics within the interview guide were based on ZB’s personal experience and research into the human caregiver experience, the interview guide used in Christiansen et al. (2013), and feedback from piloting the topic guide with eligible owners. Structured prompts were used to explore topics with owners where further depth was felt to be relevant; as the number of interviews progressed, the prompts used were increasingly based on the analysis of interviews already conducted. If owners started to move onto topics outside the scope of the interview, they were gently prompted to return to the question posed. All family members with a role in managing an eligible dog were invited to participate, and all eligible dogs within a household were discussed. Pertinent to this publication, interviews explored what owners could recall about the events leading up to their dogs’ diagnosis with osteoarthritis, and how they made initial help-seeking and treatment decisions.

### 2.2 Veterinary surgeon focus group data collection

Focus groups were chosen as a methodology to collect data from veterinary surgeons. In contrast to the owner narratives, we were interested in hearing how veterinary surgeons diagnosed and managed osteoarthritic dogs in general practice. By bringing members of the same practice together at the same time, we hoped we could create a permissive environment where ideas could be frankly exchanged and practice-wide decisions explored (Bryman, 2012). We considered mixing participants from different practices but this would have been logistically challenging and may have led to certain viewpoints being withheld. Focus groups were performed between August and December 2014 with veterinary surgeons from a sample of the general practices that had helped to recruit owners. A purposive sampling frame ensured focus groups included a range of practice sizes (single and multi-site), locations (rural, semi-urban, city) and types (independent and corporate). Practices that included staff of a range of ages and seniority levels were purposefully selected to ensure maximum diversity of experiences. Focus groups were arranged through contact with a staff member who recruited their colleagues; attendance was voluntary and participation of all eligible staff was encouraged. Details of the purpose of the study, but not specific topics to be covered, were available to participants at recruitment. Focus groups were conducted on the practice premises for convenience and to provide a safe setting for open discussion; food was provided as an incentive to attend meetings and to ease discussion (Braun and Clarke, 2013a).

All focus groups were conducted by ZB, and participants were aware of her background as a veterinary surgeon with qualitative research training. Written informed consent was obtained from all participants. For the practicalities of integrating within a working day, focus groups were limited to approximately an hour’s duration. A three-question interview schedule was used (see Supplementary Materials 1 for details). Discussion was allowed to proceed with minimal interruption and ultimately, topics covered were much broader. Additional structured prompts were used when required by the interviewer to explore topics in further depth, but typically focus group participants questioned each other’s responses such that this was not necessary. Again, the prompts used increasingly reflected initial analysis of previously conducted focus groups to explore areas of emerging interest in more detail. Since time was limited for these discussions, and the number of participants varied, some topics were explored in more depth than others in different focus groups.

### 2.3 Transcription and reflexive thematic analysis

Interviews and focus groups were recorded using a Dictaphone and professionally transcribed intelligent verbatim. Interviews and focus groups were numbered according to the order in which they were conducted, and individual veterinary surgeons were assigned numbers based on the order in which they spoke. Transcripts were not returned to participants for verification but were checked for accuracy against the audio recording. Each transcript was reviewed several times by ZB in combination with contextual field notes made during the interviews.

Thematic analysis (Braun and Clarke, 2006), now termed reflexive thematic analysis (Braun and Clarke, 2019, 2020a) was conducted by ZB, following a six-step plan described by those authors. These steps were: data familiarisation by reading the transcripts and making notes; systematic coding of the data; generating initial themes from the coded and collated data; developing and reviewing themes based on a shared meaning; refining, defining, and naming the themes; and writing the report. Analysis was undertaken using the organisational support of nVivo (nVivo v10, QSR) in parallel with data collection; constant comparison was used to ensure all opinions were included (Braun and Clarke, 2006; Coyne et al., 2014), and themes were developed by ZB from latent and semantic codes through multiple iterations of refinement. The datasets were initially analysed separately but iterative analysis developed convergent themes, as previously described by Coyne et al. (2014) and Belshaw et al. (2016a). Analysis was first conducted for the purposes of a PhD thesis (Belshaw, 2017), and continued further during the development of this, and related, publications. Reflexive thematic analysis was chosen as a methodology as: it was appropriate for the type of data collected; others within the authors’ institution had used this method so could be drawn upon for support; and since comprehensive training in the methodology was available.

Data were handled using a contextualist epistemology with an ontology based on critical realism (Braun and Clarke, 2013b) which bridges the divide between positivistic and interpretive positions (Malterud, 2016). The analysis presented acknowledges that what is said by a contributor is dependent on their own context, and how individuals experience reality depends on their culture, language and interests. This reflects a belief that a singular reality cannot be identified through qualitative research (Braun and Clarke, 2020b). Furthermore, in reflexive thematic analysis, the researcher is at the heart of the analysis and the active creation and development of the themes identified (Braun and Clarke, 2020a). This analysis therefore both tells a story and argues for the relevance of that story (Braun et al., 2019). It has been shaped by ZB’s background as a veterinary surgeon who has undertaken 12 years of UK-based clinical practice in a range of settings, her personal recollection of owning an osteoarthritic dog, and her role a researcher with a strong interest in owner experiences. Reflexive thematic analysis is distinct from ‘codebook’ qualitative research methodologies (Braun and Clarke, 2019) such as coding reliability and qualitative content analysis which typically follow a more positivist ontology. Those methods seek an objective and reliable ‘truth’ in the data through triangulation and/or consensus between multiple coders (Braun and Clarke, 2019, 2020a, 2020b).

Statistical analysis was not performed as the qualitative purposive sampling methodology captures a wide range of experiences rather than statistically representing a population (Bryman, 2012; Ziebland and McPherson, 2006). The number of participants included was considered sufficient for this exploratory research given the strict inclusion criteria, in-depth nature of the interview and focus groups, the personal experience of the interviewer as both veterinary surgeon and dog owner and the cross-case analysis performed (Malterud, 2016). Data saturation was considered to have been reached when no new themes were developed as a result of analysing additional transcripts.

Data from the same theme developed from both datasets will be juxtaposed in this report, and illustrated with quotes. Where […] is included within the quotes, it indicates that words have been removed to reduce repetition or colloquialisms; the original meaning has not been altered. Words are inserted in square brackets to preserve anonymity or improve clarity. Where two owners were involved in an interview, they are identified as interviewee ‘a’ or ‘b’.

## 3 Results

Fifty-eight owners of osteoarthritic dogs expressed interest in participating. Fifteen subsequently declined, five were unavailable during the study period, four expressed interest after the study had closed and two dogs were euthanized before interviews could be arranged. Thirty-two interviews were completed with 40 owners from 32 households who discussed 35 dogs with osteoarthritis (see Supplementary Materials 2 for owner details and Belshaw et al., 2020b for details of the dogs included). Interviews ranged 52−170 min in length.

Focus groups were conducted in four veterinary practices, involving 26 veterinary surgeons. Details of the practices and an overview of the participants in each focus group are provided in Supplementary Materials 3; to ensure anonymity, details of individual veterinary surgeons are excluded. Focus group durations ranged 51−66 min. Focus groups each included the majority of eligible participants, and all groups appeared to reflect both the demographics of their individual practices and the veterinary profession in general at that time (Buzzeo et al., 2014).

**Fig. 1:**
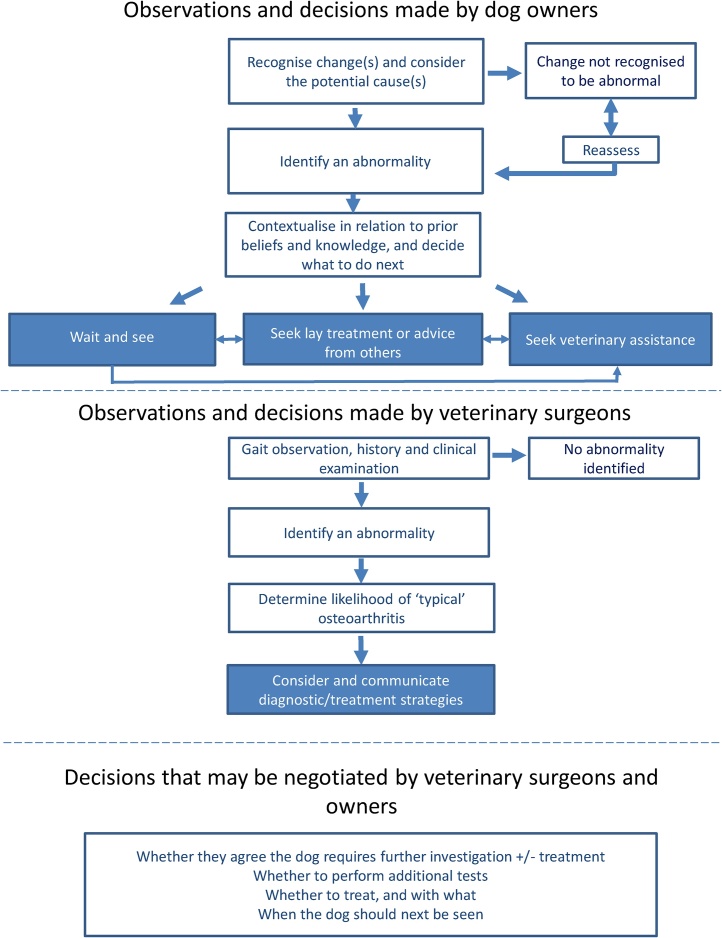
A schematic diagram summarising the key decision making steps described by owners and veterinary surgeons, individually and collectively, when deciding what to do with a dog that might have osteoarthritis.

Reflexive thematic analysis of the interview and focus group data led to the development of four themes; the theme “Could it be osteoarthritis?” is presented here, describing the initial decisions made and actions taken by veterinary surgeons and owners when a potential abnormality is recognised in a dog. The other themes described: the relationship between veterinary professionals and owners and how expertise was negotiated; how treatments and outcomes were chosen and their efficacy assessed; and the impact on the lives of owners and veterinary surgeons managing dogs with osteoarthritis. Aspects of these have been, or are planned to be, published elsewhere (Belshaw et al., 2016a, 2020a, 2020b). As an alternative way of displaying the data within this theme, the processes described are summarised in Fig. 1, based on a similar diagram in Scantlebury et al. (2014).

### 3.1 Could it be osteoarthritis? Owner narratives

#### 3.1.1 Recognition of a change

Owners typically recalled one of two behaviour changes in their dogs that signalled there might be a problem. A few dogs showed acute changes, typically non-weight bearing lameness on a single limb during or immediately after exercise, and more rarely vocalisation when moving or repeated licking of a joint or foot. This was always ascribed by their owners to the dog being in pain.

He'd chased some game into a spinney, and I thought he'd just pulled something, but it didn't improve so went to the vet. He x-rayed and diagnosed bilateral elbow dysplasia. [Interview 5]

More commonly, chronic, subtle, intermittent changes in gait and/or demeanour were identified. These included: stiffness after a long walk that typically improved through the course of a day; subtle lameness; reduced walk speed often combined with increased panting and an apparent reluctance to walk the usual distance; stumbling on a walk; reduced ability to jump into a car; increased reluctance to get out of bed or to go for a walk; reluctance to play; a change in demeanour that was interpreted by the owner as the dog being more sad or withdrawn. Pain was rarely used as a descriptor for the suspected cause of these changes. Perceived stoicism, highly variable severity of signs and long or pale fur on the limbs made it difficult for some owners to determine the significance of the problem.

It tended to always be in the evening. By the morning it seemed to have gone, you'd think 'Maybe…' So we went through several weeks if not months saying ‘She's just pulled herself slightly and she's fine now.' [Interview 23]

He is really challenging because he's very stoic. […] He's just your typical laid-back Labrador. I swear you could stick a knife in him, he probably wouldn't move. So it is difficult. [Interview 1]

#### 3.1.2 Considering the cause

Owners of younger dogs always suspected an injury as the likely cause of any change seen; several described thoroughly checking their dog’s foot for an obvious thorn or cut. In some instances, the assumption of an acute injury was correct as the dog had ruptured a cruciate ligament with concurrent osteoarthritis diagnosed radiologically or during subsequent surgery. Owners of dogs that had undergone hip or elbow scoring had sometimes discounted osteoarthritis as a potential cause since they considered their young dog unlikely to be affected.

And it was cruciate. She had damaged her cruciate. So she had it clamped. But, as it was done, the vet said “There is established arthritis already in this joint.” And she has an extremely narrow joint space on that leg. Extremely narrow on x-ray. So that was at seventeen months she had her cruciate done. [Interview 3]

I did know about hip dysplasia, and I did ask if she was hip scored, elbow tested, eye tested, and all her scores were quite low. […] She was quite a hard-core breeder, she would interview everyone. And a lot of the guys would keep in contact with her. So she said “I've never heard of anything that's come back.” So on that basis we were quite confident it wouldn’t be arthritis. [Interview 21]

Confidence amongst owners of older dogs with subtle signs that changes seen were abnormal, and that they were signs of osteoarthritis, varied widely. Ownership of multiple dogs, time spent on agility or show circuits, medical or pet health qualifications and previous dog osteoarthritis experience appeared to be associated with greater confidence in the cause. Less experienced owners typically assumed that subtle changes they saw in older dogs were part of normal ageing. Phrases such as “*I just assumed it was old age*…” and “*I just thought it was normal to slow down*…” were used by many to describe their prior beliefs. One had no idea that dogs could develop osteoarthritis.

We could tell. Labs are generally prone to arthritis. Although he'd been hip scored and all the rest of it, you can tell. My brother had a Lab, and we just know dogs because mum and dad have always had dogs. It's the most common thing. So yeah, we were expecting it to be arthritis [Interview 31]

I think probably about eighteen months ago, he was struggling getting himself up off his bed. But I just put it down to his age, and he'd gone too far in his walks and he was stiff. [Interview 2]

#### 3.1.3 Deciding what to do next

Young dogs and older dogs with acute signs were quickly presented to a veterinary surgeon. A strong bond with their dog, awareness of limits to their own knowledge and trust in their veterinary surgeon above any other information sources on pet health were other motivators for prompt veterinary attention. In contrast, many owners of dogs with subtle signs recalled adopting a ‘wait and see’ stance of regular monitoring. In some instances, this continued until the owner struggled to manage the dog’s mobility, or until they went to the practice for another reason. Assumption that little could be done for canine osteoarthritis, incorrect understanding of its pathophysiology, cost implications of a veterinary visit and concern about what the veterinary surgeon might say delayed presentation.

Yeah, he was getting up, and then he was squeaking, and then he was 'Oh yeah, I'm walking okay now.' And I am a slightly panicky owner, and I do go 'Ooh my God, that needs really investigating.' So I'll either dig at it myself, or, because there was no obvious sign, because I did check all his feet, I checked everything, it was 'Alright, get him to the vets.' I'm always happy to do more than less. [Interview 20]

I've got arthritis in this finger, and my doctor just said ‘Mm. It may hurt more now than it does eventually.’ […] But they didn't seem to be offering anything else […] I suppose I have just thought 'Well, there doesn't seem to be any magic cures for the humans’, so it never occurred to me there'd be anything more to be discussed about it from a dog point of view. [Interview 18, participant b]

Most owners had expected their veterinary surgeon to be the best available source of information about canine osteoarthritis, but typically owners recalled a paternalistic initial consultation where their dog was prescribed analgesia with little discussion about disease pathophysiology, diagnostic tests or treatment options. Few recalled asking questions during this initial consultation, even though many did not fully understand what was wrong with their dog or what the treatment might be expected to do. Rarely, owners whose dogs’ initial acute lameness was diagnosed by clinical examination as osteoarthritis had a persistent belief that an injury had been missed. Many owners subsequently sought alternative sources of information about the condition from friends, dog walkers or the internet.

I think we were just given the tablets, and that was, not quite as bad as that, but that sort of thing. I don't recall any explanation about what it was or anything like that, just getting arthritic and these are the things you need to take… [Interview 18, participant a]

Well, I've got a lot of friends who've got [osteoarthritis]. I've not got it, and me mum's not got it, so I can't really understand what it feels like, but they say all about the good days and the bad days, and things. I'm trying to ask them. 'How are you?' 'Is it painful, or is it just a nuisance?' To try and see what's happening with her. [Interview 7]

### 3.2 Could it be osteoarthritis? Veterinary surgeon focus group data

#### 3.2.1 Starting the diagnostic process

Veterinary surgeons described a process of screening almost every dog for osteoarthritis. The dog’s ability to rise and its gait walking into the consulting room were compared with a mental image of what was consistently described as ‘typical’ osteoarthritis. This ‘typical’ presentation was an older, sometimes overweight dog, that was slow and stiff on rising with mild to moderate lameness in one or more limbs. This rapid, visual assessment constituted a presumptive diagnosis to be confirmed during the consultation. The owner’s description of gait or behaviour changes were used to strengthen the likelihood of the diagnosis. Probing questions included whether the dog was stiff in the morning, and whether owners had noticed lameness or reduced willingness to exercise. For many, clinical history was the most important diagnostic aid, but all groups discussed owners who did not appear to be very observant.

… if the owner's having a pretty clear history, and you're describing typical signs of the dog struggling to get up, and possibly more lame after exercise and things like that, then often I will say 'Okay, it sounds pretty indicative.’ [Focus group 3, vet 1]

I think it very much depends. You'll have some owners who are really, really observant, and then I actually think they will often tell you almost more than your clinical exam will. But then you'll have some owners who actually don't really notice, and then your clinical exam has to be more important. [Focus group 2, vet 1]

Participants in all focus groups discussed disease ‘severity’ but were aware that how this was ascribed and recorded varied between individuals. Using joint manipulation to assess the range of motion, degree of crepitus, and associated pain appeared to be common components of a severity score. However, most veterinary surgeons found it difficult to describe exactly how these tests were used in their decision making and some disputed their relevance. No participants recalled having discussed how best to assess and record osteoarthritis severity or progression at a practice level.

I think to grade things that you can physically grade is always very nice, but arthritis degree does seem to vary from patient, from exercise level, from age, from breed. It's such a hard thing to put a grade on… [Focus group 3, vet 3]

And also what difference is [grading the disease] really going to make to the dog? It’s better on [meloxicam]; it’s not better on [meloxicam] but is on tramadol. Is that bad to say that? But it is true. Okay, so it was a five and now it’s a three. So? [Focus group 1, vet 1]

#### 3.2.2 Confirming the diagnosis

Attitudes to radiography varied within and between practices. Most veterinary surgeons used only history and clinical examination when diagnosing ‘typical’ osteoarthritis, suggesting confidence at the signs’ specificity. A few routinely offered radiography but others left it for the owners to suggest. Several thought radiographs did not provide additional useful diagnostic information in ‘typical’ osteoarthritis as it rarely changed what they did. However, in a young, acutely lame dog or with a rapid increase pain or lameness in an older dog, participants generally agreed that they would advise radiography to rule out other differential diagnoses.

I think people that come in with a lame animal… you can tell those clients that want to do more, can't you… and then you could say 'Look, we can x-ray, there's no problem with that.' Because it rules out other things, doesn't it, it gives them peace of mind that it is just arthritis. I'm not particularly proactive about that. [Focus group 2, vet 1]

With the older dog probably we advise x-ray if it doesn’t improve after maybe a month – or you x-ray first to diagnose osteoarthritis in the first place to make sure it’s nothing sinister. A young dog is a mandatory x-ray after a week if it’s not improved. [Focus group 1, vet 1]

Most participants did not think force plate gait analysis, arthroscopy, additional gait observation or paper-based owner outcome measures were necessary or practical in a general practice setting. Time was a consistent perceived barrier, even with a designated consultation length of 15 min. For many, the final piece of evidence needed to confirm the diagnosis of ‘typical’ osteoarthritis was response to treatment, typically with a non-steroidal anti-inflammatory drug (NSAID). At the end of a 5–14 day period, history and a repeat clinical examination were used to confirm a response. ‘Typical’ osteoarthritis was expected to respond well to NSAIDs in almost all cases.

We should be analytical and using some pain scoring system or getting the owner to fill in questionnaires; but that’s just not what 15-minute consultations allow you to do. That is what would be the best if it was recorded, but it doesn’t fit. In general practice you’ve got to go with your hunches and go with what the owner says. [Focus group 1, vet 2]

[The owners] come back and then they go 'Wow, they're a completely different dog.' [Focus group 4, vet 3]

#### 3.2.3 Persuading owners to instigate treatment

Most veterinary surgeons expected owners to know that dogs developed osteoarthritis. Consistently, two owner attitudes to the diagnosis were described. The first was denial, where owners were perceived to respond to questions with replies that implied they did not want to discuss the topic. The second attitude was more open, inviting further explanation. These initial attitudes were perceived by participants to predict how the rest of the consultation might progress. Vaccination consultations were discussed as a particularly challenging time to diagnose osteoarthritis as time was tight and osteoarthritis was not the primary reason the owner had presented the dog. Rarely, veterinary surgeons decided not to mention that the dog might have osteoarthritis rather than attempt to fit it into such consultations.

You do get the odd one who takes it really badly, and cries. And you say 'Well no, no, at most the dog's a bit sore, and you just have to manage it.' And then they take some management, and some of them are the dogs that aren't actually that bad. So where they've been hiding… I don't know where they live that they've not heard of arthritic dogs before. [Focus group 4, vet 1]

I think any vaccination appointment which is time-limited to a clinical examination history, and administering vaccination, sorting worming, parasitic therapies, etcetera. I think if you discover any significant disease […] it's a really difficult thing to keep to schedule. If you're consulting on your own, or with another person, and you don't want to drop them in it by you being very thorough and doing a 25 min consultation. [Focus group 3, Vet 5]

There was broad consensus that the most effective means to convince owners of a problem was trial treatment. Some veterinary surgeons relied on owners noticing improvement with treatment, whilst others withdrew NSAIDs after the trial period to demonstrate the difference. All veterinary surgeons compared “*strategies”* or “*tactics*” to “*persuade*” or “*convince*” resistant owners to trial some analgesia. These included demonstrating changes and pain responses on clinical examination and exercising the dog outside the practice premises to demonstrate lameness. Several suggested these strategies rarely helped and there was broad agreement that owners left the consulting room unconvinced. This was a source of major frustration, and veterinary surgeons considered these owners as a barrier to the dog receiving treatment.

Sometimes even just the physical exam and the dog turning around and being clearly uncomfortable when you’re manipulating a joint they go, “Oh actually maybe there is a pain thing there; they’re not just old and stiff”. [Focus group 3, vet 5]

And I think when you say 'The last thing I would want is for your animal to be in any discomfort and we haven't realised.', I think also they think 'Oh, that's true.' And then they're a bit more open to thinking 'Right, well, it's only a week, let's just try it. It keeps the vet quiet.' [Focus group 3, vet 3]

## 4 Discussion

This qualitative research describes the pathways followed by osteoarthritic dogs from first recognition of a clinical change by owners to diagnosis and management in a general practice setting. By juxtaposing accounts from owners and veterinary surgeons, we identify motivators and barriers to osteoarthritis diagnosis and demonstrate the value of exploring the experiences, attitudes and opinions of the people on both sides of the consulting room table, as summarised in Fig. 1. Initial behavioural signs of canine osteoarthritis may be more wide ranging and subtle than those previously described, and poor awareness of their cause amongst owners may delay veterinary presentation. Short consultation lengths, inaccurate and unrealistic expectations of owner knowledge, challenges interpreting the cause of clinical signs and difficulty formulating effective negotiating strategies may limit general practitioners’ ability to treat canine osteoarthritis. Expert guidance on how to diagnose canine osteoarthritis does not appear to be followed in general practice, rather a truncated diagnostic pathway appears to be widespread, the sensitivity and specificity of which has yet to be determined. These findings demonstrate the need for practical evidence-based guidance for the recognition of canine osteoarthritis by owners and for its diagnosis by general practitioners.

The decision-making processes described by dog owners in this study has parallels to findings from human medical sociology. Helman (1981), describes individual patients and family groups as constructing ‘folk models of illness’; personal reasonings behind what might be happening and why, what will happen if they do nothing, and what might be done for a specific illness or symptom set. These same types of personal reasons are evident from the responses of our dog owners. Helman describes ‘folk models’ as leading to three broad outcomes for health decisions: wait and see, lay treatment including asking others for advice, or seek help from a healthcare professional. Such relationships between beliefs and outcome options are also evident in our data and in a model of equine colic treatment (Scantlebury et al., 2014), and support the inclusion of these factors in the theoretical Pet Care Competency model (Glanville et al., 2020). The perceived impact of a diagnosis has also been found to affect whether a person visits a general practitioner. Strong feelings of fear and death associated with cancer can lead to avoidance of a consultation with a general practitioner, despite recognising relevant symptoms (Sheik and Ogden, 1998). Similar negative associations are described by owners in relation to canine osteoarthritis (Belshaw et al., 2020b), and the fear of confirming something they believe to be associated with a negative may play a role in delayed presentation. This study supports the presence of analogous ‘folk models of illness’ for pet owners, perhaps better termed ‘lay models’, which may influence their behaviour in predictable ways. Decision making around early behavioural changes is clearly complex and should be further explored to better tailor communications with pet owners.

Published behavioural changes associated with canine osteoarthritis include: inactivity stiffness, limping or lameness, reduced willingness to jump up and/or down, willingness to play, exercise intolerance, aggression, vocalisation, and postural shifting (Belshaw et al., 2016b; Brown, 2014; Cachon et al., 2018; Hielm Bjorkman and Tulamo, 2008; Innes, 2018; Pettit and German, 2015; Walton et al., 2013). The previous most wide-ranging list of behavioural changes was generated through focus groups with 30 owners of osteoarthritic dogs affected in a single proximal joint (Brown, 2014). Owners in that study described their dogs stopping on walks, hesitating before making big movements and reducing their normal activities. Our findings include responses from owners of dogs with osteoarthritis in multiple joints who typically first identified a wider range of chronic, subtle and intermittent changes in gait, pace and/or demeanour. Veterinary surgeons in focus groups in this study also reported using an improvement in demeanour as a sign of effective analgesic treatment. These signs are unlikely to be disease-specific, but appear consistent. Describing behavioural changes that owners notice and interpret may better support recognition of the early signs of canine osteoarthritis and improve communication between owner and veterinary surgeon.

Both veterinary surgeons and owners expressed uncertainty in knowledge of the behavioural changes associated with pain or osteoarthritis severity. Recognising canine chronic pain is difficult (Bell et al., 2014; Belshaw and Yeates, 2018; Sharkey, 2013). In people, osteoarthritic pain is complex and highly variable with fifteen unique types of osteoarthritis pain described (Cedraschi et al., 2013). ‘Flare ups’ where pain suddenly intensifies, are well described, yet poorly understood in human osteoarthritis (Thomas and Neogi, 2020). Given the pathophysiological similarities between human and canine osteoarthritis (Meeson et al., 2019), a similarly extensive range of pain morphologies could be experienced by dogs. This may go some way to account for the wide range and intermittent nature of behavioural changes owners describe, as may the recent finding that owners and veterinary surgeon perceive there to be breed differences in pain experience (Gruen et al., 2020). Educating owners about the wide range of behavioural signs to look for and their potential relationship with pain may decrease the time to initial veterinary presentation and since this research was conducted, a wide-ranging awareness campaign for owners has been launched (Canine Arthritis Management, 2020).

Veterinary surgeons who took part in our focus groups appeared confident that thorough history taking, clinical examination and a positive response to treatment constituted a sufficiently sensitive and specific diagnostic pathway for ‘typical’ canine osteoarthritis. In contradiction to the expert-led diagnostic pathways in published literature (Cachon et al., 2018; Innes, 2012; Pettit and German, 2015), diagnostic tests were rarely used where the clinical picture matched expectations. This mirrors observational data that found around 70 % of consultations undertaken in general practice did not lead to any diagnostic tests (Robinson et al., 2016). Lack of time and uncertainty as to additional benefits further tests might confer were cited as barriers to performing more tests in the current study, and the importance of pragmatism in general practice was raised. Similar pragmatic clinical reasoning and uncertainties behind the choice of diagnostic tests have been described amongst human medical general practitioners (Gabbay and le May, 2016; Ingvarsson et al., 2020). The consistency of the diagnostic pathway described by our participants could be tested in a larger group of veterinary surgeons using vignette examples, similar to recent research by Guervara et al. (2019).

Convincing some owners to treat their dogs for canine osteoarthritis appears challenging, and deeply frustrating for veterinary surgeons. This apparent reluctance to treat carries a multitude of risks to the welfare of those dogs, and to the relationships of trust between owners and veterinary surgeons (Corah et al., 2019). Short consultation lengths, unrealistic expectations of owner knowledge, and difficulty formulating effective negotiating strategies were barriers these veterinary surgeons recognised and that have been discussed elsewhere (Armitage-Chan and May, 2018; Belshaw et al., 2016b, Belshaw et al., 2018a; King et al., 2018; McDermott et al., 2015, 2017; PDSA Animal Wellbeing Report, 2018). Whilst they did not directly reflect upon it, some veterinary surgeons also appeared not to view themselves as having an educational role; this too has previously been identified in relation to vaccination consultations (Belshaw et al., 2018a). The impact of owner prior beliefs may play a previously unidentified role in the apparent reluctance of some to accept veterinary recommendations. Helman (1981) cautions on the resistance to change of ‘folk models’ and Hunt et al. (1989) found women took a long time to incorporate information from a general practitioner into their beliefs. This suggests that it may be unrealistic to expect dog owners to change their prior beliefs about the cause and significance of the signs they have noticed after a single consultation, and that exploration of existing ‘lay models’ and assumptions during the consultation may be useful. Consultation models that acknowledge the existence and importance of prior beliefs, such as the Two Houses model (Brunet, 2020), may be key in building trust and improving these interactions.

It is important to acknowledge the limitations of this work. Our purposive samples were designed for qualitative research so these findings should not be statistically/probabilistically extrapolated (Smith, 2018). However, we hope that naturalistic generalizability has been achieved, whereby the contents of this publication intuitively resonate with the tacit experiences of the reader (Smith, 2018), whether clinician or dog owner. As described above, many of our findings also fit closely with previously published work in this field. The nature of reflexive thematic research means this work was shaped and developed primarily by the lead author; others may have developed different themes and presented these data differently, but that does not reduce the validity or importance of this work. Interview and focus group locations were restricted to England and Scotland due to time and funding. The impact of this limitation is unclear; it is unknown whether geographic location affects experiences or attitudes in this context. Hindsight and recall bias may have occurred when participants described past events, and their accounts will have been shaped by the context and timing of the interviews and focus groups, and the questions and prompts used. A definitive diagnosis of canine osteoarthritis was not a criterion for inclusion. This was appropriate since a review by Belshaw et al. (2016b) identified over 600 methods characterising canine osteoarthritis with no consensus on how “definitive” might be defined. In addition, the low prevalence of definitive diagnoses made in UK veterinary practices (Robinson et al., 2016) would have excluded many dogs. The focus group methodology may have led some veterinary surgeons to withhold certain opinions in front of colleagues, but this was mitigated, as much as possible, by creating boundaries that identified focus groups as a safe space. In addition, since focus groups were time limited, veterinary surgeons’ decision making could not be explored in such depth as was possible with owners. The figure provided attempts to reflect the data included in this publication in a user-friendly way, and as such some of the detail contained in the narratives and focus group data cannot be included. Nevertheless, we believe it to be a useful illustration which future research can build upon.

## 5 Conclusions

Canine osteoarthritis is common, painful and can have severe impacts on the welfare of both dogs and their owners (Belshaw et al., 2020a, b). This research indicates that decision-making to reach a diagnosis and instigate treatment can protracted, complex, negotiated and may be deeply frustrating. The early signs of canine osteoarthritis can be subtle, and change may be very gradual or intermittent. Owners’ prior beliefs about the cause of changes seen, and about canine osteoarthritis as a diagnosis, may shape their decisions and influence the impact of information exchanged during a consultation. Veterinary surgeons rarely seem to follow the expert-led advice on osteoarthritis diagnosis, and the efficacy of alternatives described here should be further investigated. Short consultation lengths appear to present an ongoing challenge to successful consultations. Ultimately, this research demonstrates that both people in the veterinary consulting room want the dog under discussion to be happy and comfortable. Our data identifies many new avenues for future research and improved communication strategies that could facilitate earlier identification of canine osteoarthritis, enabling this shared goal to be fulfilled.
