# Supplementary material for: Could it be osteoarthritis? How dog owners and veterinary surgeons describe identifying canine osteoarthritis in a general practice setting
Source: Prev Vet Med. 2020 Dec;185:105198. doi: 10.1016/j.prevetmed.2020.105198 (PMC7755036; doi:10.1016/j.prevetmed.2020.105198)
Supplement: Supplementary file 2 [file mmc2.docx]

**Supplementary materials 1. Focus group question schedule and potential prompts (other ad hoc prompts were used as relevant)**

1. How do you diagnose osteoarthritis in dogs?

a) Is it more common in specific consultation types?

b) Do you use any diagnostic tests? How do you decide?

c) Is that always what you do or does it vary?

d) Do you use any kind of scoring for severity?

d) Do you think owners recognise the problem? How do those conversations go? What do they know about the condition?

2. How do you typically manage a dog with osteoarthritis?

a) Do you have any practice protocols? If yes, were you all aware of that and is it what you always do?

b) Does the age of the dog make any difference, or any comorbidities?

c) Are you concerned about adverse events? Do you do any routine checks?

d) What about supplements and other therapies like hydrotherapy? How confident are you with those?

e) How do you find getting owners on board – is it relatively easy?

3. What do you do during a re-check?

a) Do you always do a clinical exam? How does that fit with what owners say?
 b) What do you change based on what you’ve seen?

c) Do you use any kind of monitoring tool?

d) How do you decide when to change medications?

**Supplementary materials 2. Details of owners included in the interview study**

| **Interview number** | **Interviewee sex(es)** | **Estimated interviewee age(es)** | **Interview location region** | **Owner house location** | **Household status** | **Children living at home** | **Previous**  **dog owning experience as an adult?** | **Previous dog osteoarthritis experience?** | **Currently own more than one dog?** |
| --- | --- | --- | --- | --- | --- | --- | --- | --- | --- |
| 1 | F | 36-65 | Midlands | Rural | Cohabit | N | N | N | Y |
| 2 | F | 36-65 | Midlands | Urban | Single | Y | Y | Y | N |
| 3 | F | 36-65 | Midlands | Conurbation | Cohabit | N | Y | Y | Y |
| 4 | F | 36-65 | Midlands | Rural | Cohabit | N | Y | Y | Y |
| 5 | F | 36-65 | Midlands | Urban | Cohabit | N | Y | N | N |
| 6 | F | 18-35 | Midlands | Rural | Cohabit | N | Y | N | N |
| 7 | F | 36-65 | Midlands | Conurbation | Cohabit | N | Y | N | N |
| 8 | F, M | 66+, 66+ | Midlands | Urban | Cohabit | N | Y, Y | N, N | Y |
| 9 | F | 18-35 | East Anglia | Rural | Cohabit | N | Y | N | Y |
| 10 | F | 36-65 | South East England | Urban | Cohabit | Y | N | N | N |
| 11 | M | 36-65 | South East England | Urban | Cohabit | Y | Y | N | N |
| 12 | F, M | 36-65, 66+ | South East England | Urban | Cohabit | N | Y, Y | Y, N | N |
| 13 | M | 66+ | South East England | Urban | Single | N | Y | N | N |
| 14 | F | 36-65 | South East England | Rural | Single | N | Y | N | N |
| 15 | F, M | 36-65, 36-65 | South East England | Urban | Cohabit | Y | Y, Y | N, Y | N |
| 16 | F | 36-65 | South East England | Urban | Single | N | Y | N | N |
| 17 | F, M | 66+, 66+ | South East England | Conurbation | Cohabit | N | N, N | N, N | Y |
| 18 | F, M | 66+, 66+ | South East England | Rural | Cohabit | N | Y, Y | N, N | Y |
| 19 | F | 36-65 | East Anglia | Rural | Cohabit | N | N | N | Y |
| 20 | F | 36-65 | East Anglia | Rural | Cohabit | N | Y | Y | Y |
| 21 | F | 36-65 | East Anglia | Rural | Cohabit | N | Y | N | Y |
| 22 | F | 36-65 | East Anglia | Rural | Cohabit | N | N | N | N |
| 23 | F | 36-65 | Southern England | Urban | Cohabit | Y | N | N | N |
| 24 | F, M | 66+, 66+ | South West England | Urban | Cohabit | N | Y, Y | Y, Y | N |
| 25 | F, M | 66+, 66+ | South West England | Urban | Cohabit | N | Y, Y | Y, Y | Y |
| 26 | M | 36-65 | Central Scotland | Conurbation | Single | N | Y | Y | N |
| 27 | F | 36-65 | Central Scotland | Conurbation | Single | Y | Y | Y | N |
| 28 | F | 66+ | Central Scotland | Conurbation | Cohabit | N | Y | N | N |
| 29 | F | 36-65 | Central Scotland | Conurbation | Cohabit | Y | N | N | N |
| 30 | M | 36-65 | Central Scotland | Conurbation | Cohabit | Y | N | N | N |
| 31 | F, F | 36-65, 66+ | Central Scotland | Conurbation | Cohabit, single | N | Y, Y | N, N | N |
| 32 | F | 36-65 | Central Scotland | Conurbation | Cohabit | N | N | N | N |

**Legend**: N = no, Y = yes, F = female, M = male

**Supplementary materials 3. Details of the veterinary practices in which focus groups were performed.**

| **Focus group number** | **Focus group type** | **Practice description** | **Approximate number of veterinary surgeons/veterinary nurses eligible for inclusion (data from practice websites)** | **Total number of participants (Number of males)** | **Number of practice partners eligible for inclusion (attending)** | **Length of standard consultation** | **Participant description from contemporaneous field notes and information on the practice website where available** | **Timing of focus group** |
| --- | --- | --- | --- | --- | --- | --- | --- | --- |
| FG1 | Veterinary surgeon | Multi-branch, city, small animal private practice | 15 | 11 (4) | 3 (3) | 15 minutes | Range of veterinary surgeons from a new graduate to senior partners. All permanent staff; most had worked at the practice for several years. Most worked in multiple branch practices. | Evening |
| FG2 | Veterinary surgeon | Single branch, town, small animal private practice | 5 | 4 (0) | 2 (1) | 10 minutes | Three permanent veterinary surgeons, one regular locum. All had been involved with practice at least 5 years. The permanent veterinary surgeons had all worked at this practice for many years. | Lunchtime |
| FG3 | Veterinary surgeon | Multi-branch, city and town small animal private practice | 20 | 6 (2) | 5 (1) | 10 minutes | Five permanent vets within their first 10 years since graduation, only one of whom had been at the practice for several years plus one senior veterinary surgeon who had been at the practice many years. All worked in multiple branches. | Lunchtime |
| FG4 | Veterinary surgeon | Multi-branch, village, mixed, private practice | 5 | 5 (0) | 2 (1) | 10 minutes | Range of veterinary surgeons: one new graduate, two graduates of fewer than 10 years since graduation and two senior partners who had been at the practice many years. One veterinary surgeon also did equine work. All worked in branch practices. | Evening |
